# Mechanical Properties of Vacancy Tuned Carbon Honeycomb

**DOI:** 10.3390/nano9020156

**Published:** 2019-01-27

**Authors:** Lu Xie, Haojie An, Chenwei He, Qin Qin, Qing Peng

**Affiliations:** 1 School of Mechanical Engineering, University of Science and Technology Beijing, Beijing 100083, China; xielu@ustb.edu.cn (L.X.); anhaojie@xs.ustb.edu.cn (H.A.); 2 Reactor Engineering and Safety Research Center, China Nuclear Power Technology Research Institute Co., Ltd., Shenzhen 518031, China; hechenwei@cgnpc.com.cn; 3 Nuclear Engineering and Radiological Sciences, University of Michigan, Ann Arbor, MI 48108, USA

**Keywords:** carbon honeycomb, molecular dynamics, defect, mechanical properties

## Abstract

Carbon honeycomb (CHC) has great application potential in many aspects for the outstanding mechanical properties. However, the effect of both defects and temperature on the mechanical properties are far from reasonable understanding, which might be a huge obstacle for its promising applications as engineering materials. In this work, we investigate the effect of vacancy-type defect, which is inevitably exists in material, on the mechanical properties of CHC via reactive molecular dynamics simulations. The mechanical strength is anisotropic and decreases with the increasing temperature. CHC yield in cell axis direction since the break of C–C bonds on the junction. Vacancies weaken CHC by reducing the strength and failure strain. The effect of single vacancy on strength of CHC becomes more obvious with reducing temperature and is sensitive to the location and bonding of the vacancies. The maximum reduction of strength in cell axis direction is with vacancy on the middle of the wall of CHC where sp^2^ bonds are removed. The strength is reduced by 8.1% at 500 K, 11.5% at 300 K and 12.8% at 100 K. With 0.77% defect concentration, the strength reduces 40.3% in cell axis direction but only 18.7% in zigzag direction and 24.4% in armchair direction.

## 1. Introduction

Carbon is one of the most versatile and flexible elements, which can form a variety of carbon allotropes including both 1D carbon nanotubes (CNTs) and 2D graphene with different atomic arrangements. Both CNT [1,2] and graphene [3] have been reported with superb mechanical and thermal properties. However, it is difficult to get engineering practices, because such properties are significantly lower than those of monolayer graphene [4] or CNTs [5,6] when scaling up to 3D structure. Therefore, efforts have been shifted to the design of 3D carbon materials. With unique 3D honeycomb structure CHC is not limited by this problem, having broad application prospects. CHC proposed by Park et al. [7] has excellent mechanical and thermal properties and it can be used not only for storage of gases and liquids but also as a matrix for new composites [8,9].

There is a huge interest in the atomic-level structure and performance of CHC since the first introduction. The first stable CHC is obtained by deposition of vacuum-sublimated graphite by Nina V. Krainyukova et al. [10] in 2016. CHC with sp^2^–sp^3^ hybridization has a high structural stability at low mass density and high porosity [11]. Based on the CHC structure, Chen et al. [8] demonstrate the unusual magnetic transport properties in the CHC nexus networks. Shuaiwei Wang et al. [9] propose new families of stable semi-metallic CHC structure with multiple Dirac cones, high Fermi velocity and high porosity. The electronic band structure and electronic density of CHC indicate a similar size dependence as for carbon nanotubes [11]. CHC is also found to have a high thermal conductivity, about 100 W/mK along the honeycomb axis direction [12]. And the thermal conductivity parallel to the honeycomb axis direction is one order of magnitude higher than the perpendicular direction [13]. 

3D CHC has promising applications in many aspects, which is related to its mechanical properties. For example, the local buckling [14] is more benefited to the transportation of the absorbed gas molecules inside CHC rather than the global buckling. Therefore, it is indispensable to study the mechanical properties of CHC. There are lots of studies about the mechanical behavior of CHC. Jin Zhang et al. [14] studied the buckling of CHC under uniaxial compression by molecular dynamics (MD) method and CHC exhibits two topographically different buckling modes when subjected to the uniaxial compression in the armchair and zigzag directions. In particular, the nonlocal effect originating from van der Waals interactions greatly reduces the ability of CHC to resist structural instability and leads to early onset of CHC buckling. Zhenqian Pang et al. [12] discussed the specific strength of carbon honeycombs with different crystal cell sizes and studied the influence of size and direction on Poisson’s ratio by equilibrium MD simulations. The mechanical properties of CHC studied by Zhang et al. [15] show that the Young’s modulus of the structures is determined solely by the density of the hinges, regardless of the structural orientation or regularity and the failure strain of the honeycomb structure is affected significantly by its lattice size and geometrical regularity. Gu et al. [16] studied the effects of carbon atoms in the triple junction on the performance of CHC and discussed the mechanical properties of CHC with different chirality and sizes. In short, the mechanical properties of CHC has strong lattice size effect and direction dependence. The failure strain and failure decrease with decrease the size of crystal cell of CHC [12]. 

Mechanical properties of a material are often associated with defects. Vacancy defects resulting from missing carbon atoms likely occur in synthesis process. Vacancy defects have already been detected in carbon materials including both CNTs and graphene. Single atom vacancy defect is reported to reduce the strength of CNT by 19% [17]. The failure strength of defective graphene is 14% smaller than pristine one [18]. There is more reduction in strength with the increasing defect concentration. But little is known about defects that might by introduced during CHC synthesis. Currently, the tensile behavior of CHC with particular size is not clear and the effect of defect on mechanical properties of CHC is not yet known. 

In this paper, a CHC with graphene as the basic unit is constructed. MD method has been employed to study the mechanical properties of CHC along different directions. The effect of single atom vacancy defect on mechanical properties of CHC in different directions are discussed systematically. The influence of temperature and defect concentration has also been investigated.

## 2. Materials and Methods

**Figure 1:**
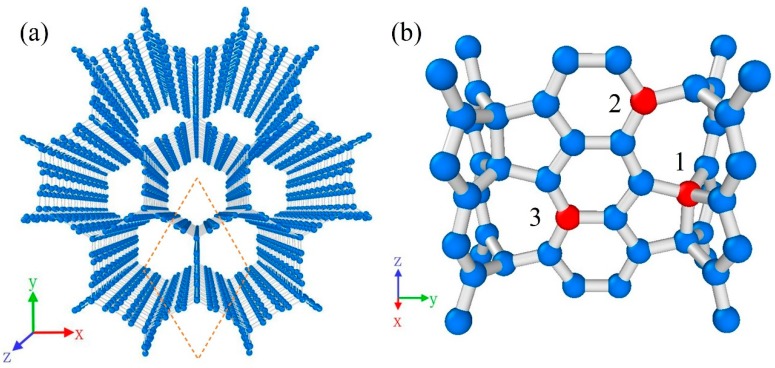
Snapshots of carbon honeycomb. (**a**) Perspective view of atomic structure of CHC; (**b**) Atomic structure on the junction. Red atoms represent three kind of vacancy defects of CHC.

The atomic structure of CHC is shown in Figure 1a. It can be viewed as consisting of zigzag-edged graphene nanoribbons with sp^2^ bonding in the wall and sp^3^ bonding in the junction. The cross-section perpendicular to cell axis is a honeycomb structure based on regular hexagon with a side length of 5.8 Å. The junction is composed of an array of cell units containing two 5-rings and one 8-ring, as shown in Figure 1b. 

MD method is widely used in many fields [19,20]. Here, Classical MD simulations are adopted to study the tensile behaviors by the Large-scale Atomic/Molecular Massively Parallel Simulator (LAMMPS) package [21], while the structural analysis and post-processing are performed with OVITO [22]. The atomic interactions for C–C are described by the adaptive intermolecular reactive empirical bond order (AIREBO) potential with modified C–C bond cutoffs [23], which can be used to describe the thermal and mechanical properties of CHC at large strains [24]. All MD simulations are simulated at the isothermal-isobaric (NPT) ensemble with periodic boundary conditions in all the directions. Velocity-Verlet algorithm is employed to integrate Newton’s equations of motion with a time step of 0.5 fs. The temperature and pressure are controlled using Nose Hoover thermostat and barostat with coupling times for set at 0.025 fs and 0.25 fs, respectively. 

Firstly, conjugate gradient algorithm is performed so that CHC is relaxed to a minimum energy state. Then a stable CHC structure with 12,906 atoms is obtained by relaxing at room temperature (300 K) and zero external pressure for 25 ps. The dimensions of the CHC structure are 59.43 Å × 51.51 Å × 57.87 Å. Finally, tensile tests have been performed by expanding the box size along specific directions (zigzag, armchair and cell axis, respectively) at engineering strain rate of 10^9^ s^−1^. The data of stress and strain is output every 0.25 ps.

## 3. Results and Discussion

### 3.1. Mechanical Properties

**Figure 2:**
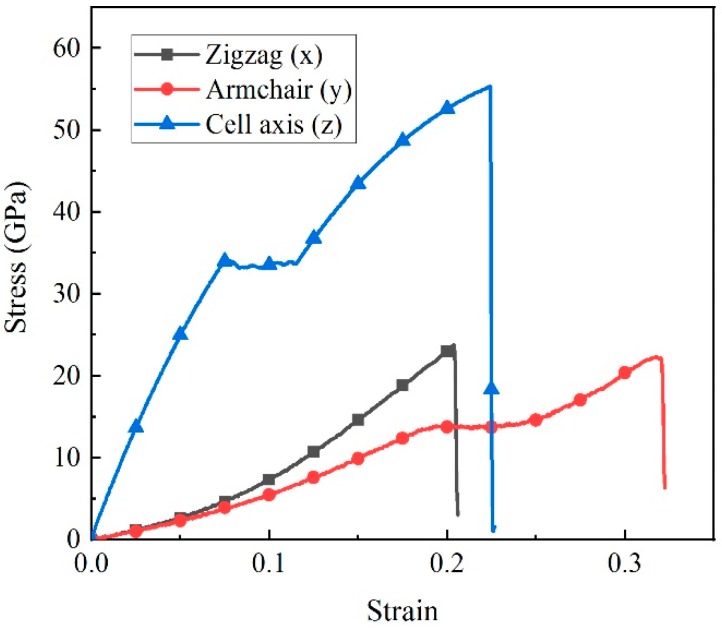
Engineering stress-strain curves of carbon honeycomb for tensile loading along zigzag, armchair and cell axis direction, respectively.

Figure 2 shows the engineering stress-strain curves along zigzag (x), armchair (y) and cell-axis (z) directions, respectively. Note that the engineering strain *ε*_x_ by definition is calculated as

*ε*_x_ = (*L*_x_ − *L*_x0_)/*L*_x0_ (1)

with *L*_x_ and *L*_x0_ as the deformed and initial CHC lengths in the x direction. One sees from Figure 2 that the Young’s moduli and tensile strength along cell axis direction are bigger than that along the other two directions at tensile tests of CHC structure. At the beginning of the tensile test, the stress increases near linearly with the increase of strain. Then CHC yield at 0.075 tensile strain with yield strength 34 GPa. Harding stage are observed after the strain increased to 0.115 and the ultimate tensile strength is up to 55.3 GPa with failure strain 0.225. Finally, the stress-strain curve drops suddenly, indicating the failure of the nanostructures. The same trend is detected in the stress-strain curve when stretched along the armchair direction. However, for loading along zigzag direction, the stress growth accelerates accompanying the increasing of tensile strain. The strengths are similar when tensile strain applying along zigzag or armchair direction.

To further understand mechanical properties of the CHC structure, Young’s modulus, failure strains and tensile strength are listed in Table 1. The CHC structure with side length 5.8 Å has a Young’s moduli 550 GPa along cell-axis direction, which is about an order of magnitude higher than that along the other two directions and a tensile strength 55.3 GPa along cell-axis direction, which is about two times as much as in other two directions. The mechanical strength is consistent with density functional theory (DFT) calculations [12]. In addition, the failure strain is up to 0.320 along armchair direction. 

**Figure 3:**
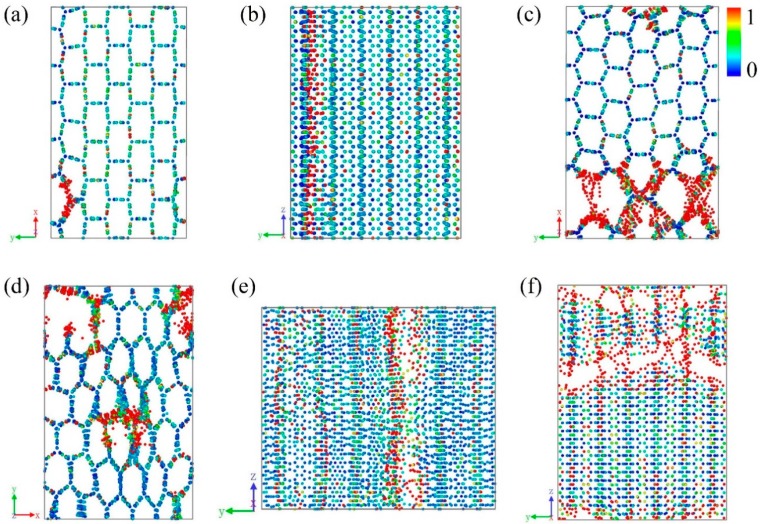
Shear strain. (**a**–**c**) Evolution of local shear strains for loading along zigzag direction. (**d**–**e**) Evolution of local shear strains for loading along armchair direction. (**f**) Distribution of local shear strains for loading along cell axis direction.

The fracture of a material corresponds to the irreversible motion of some atoms. Here, the von Mises strains (scales from 0 to 1) has been employed to study the local atomic strains by using OVITO with cutoff radius of 3.5 Å. Figure 3a–c exhibit the evolution of local shear strains for loading along zigzag direction. The shape of the CHC hole changes from regular hexagon to rectangle and stress is evenly distributed over the wall parallel to tensile direction before failure. Failure initiated from the C–C bond that is parallel to tensile direction. The evolution of local shear strains shows that fracture occurs by rapid crack propagation along cell axis direction and then along armchair direction. Figure 3d–e shows the evolution of local shear strains along armchair directions. Similarly, failure propagates in the cell axis direction and then in the zigzag direction. Figure 3f shows the distribution of local shear strains when tensile in cell axis direction. The crack triggered from a certain position propagates in multiple branches. The fracture surface of CHC is consistent with CNT or graphene [25] but since the unique 3D structure CHC exhibits different crack propagation patterns.

### 3.2. Effect of Vacancy Defects on Mechanical Properties

Generally, point defect within nanomaterials is inevitable in the process of synthesis. Here, the mechanical properties of CHC with vacancy defect have been investigated. When removing an atom from CHC, there could be some different conditions. A point vacancy on the junction and two kind of point vacancies on the wall parallel to armchair direction have been considered. Each carbon atoms around the defect is saturated so that the structure is stable. Figure 1b illustrates the position of the three point-defects. Defect 1 is a vacancy on the junction of CHC. Defect 2 and 3 are vacancy defects on the wall of CHC. Meanwhile, defect 2 is a kind of point vacancy near the junction.

**Figure 4:**
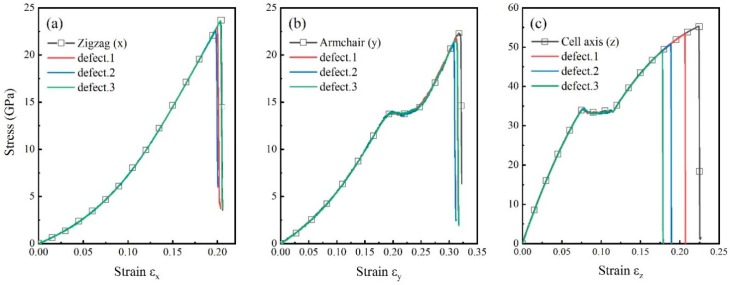
Effect of vacancy defect. Engineering stress-strain curves of carbon honeycomb for loading along (**a**) zigzag, (**b**) armchair and (**c**) cell axis directions, respectively.

Figure 4 shows the engineering stress-strain curves of pristine and vacancy defected CHCs along three different directions. The curves with point-defects coincides with the pristine curve when tensile strain is less than failure strain, implying that single atom vacancy has little influence on Young’s moduli. The main reason for CHC breaking along zigzag direction is the failure of C–C bond on the wall parallel to this direction. So little reduction on tensile strength has been detected. The strength of CHC with different vacancy defects ranges from 22.6 to 23.6 GPa. The strength difference between defected and pristine CHCs in x direction is within 5% (Figure 4a). For tensile loading along armchair direction (Figure 4b), the maximum tensile strength with vacancy defects is 21.2–21.8 GPa, that is around 5% smaller than pristine one. Figure 4c shows the stress as a function of tensile strain in cell axis direction. The tensile strength is ranging from 48.9 to 53.3 GPa. There is a 11.5% reduction in tensile strength along cell axis direction with defect 3. Vacancy defect weaken CHC by reducing the tensile strength and the reduction of tensile strength in cell axis direction is larger than that along the other two directions. 

**Figure 5:**
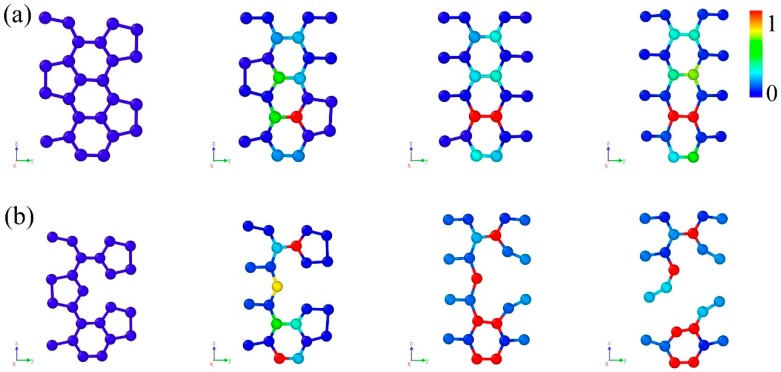
Evolution of local shear strain for loading along cell axis direction. (**a**) Pristine CHC. (**b**) CHC with defect 3.

The result in Figure 4c shows that the position of vacancies is critical for the strength of CHC in cell axis direction. The maximum reduction of tensile strength of CHC is along cell axis direction with defect 3. Figure 5a shows the evolution of local shear strains and deformation of pristine CHC. Meanwhile, the middle two columns are the atoms on the wall and other atoms are on the junction of CHC. At the tensile process, atoms on the wall are subjected to larger shear strain than atoms on the junction of CHC. This explains why the influence of defect 2 and defect 3 (vacancy on the wall) on the strength of CHC in cell axis direction is greater than defect 1 (on the junction). 

To further understand the effect of vacancy defects on CHC, the total energy of both pristine and vacancy defected CHCs and the formation energy of vacancy defects are calculated by MD simulations. The calculation detail is documented in the Supplementary Files. Results show that the energy per atom is −7.24 eV, which is consistent with the first-principles density functional theory calculations [26]. It is lower than vacancy defected CHCs (−7.22, −7.21 and −7.19 eV for CHC with defect 1, defect 2 and defect 3, respectively). The calculated formation energies of vacancy defects are 12 eV for defect 1, 14 eV for defect 2 and 18 eV for defect 3. The formation energies of defect 1 with sp^3^ hybridization is lower than both defect 2 and 3 with sp^2^ hybridization. Therefore, it is reasonable that the order of mechanical strength of CHC is pristine > defect 1 > defect 2 > defect 3 in cell axis direction.

Otherwise, C–C bonds formed by atoms on the junction (5-ring) break as shown in Figure 5a. There are sp^2^ hybridized carbon atoms on the wall and sp^3^ hybridized carbon atoms on the junction of CHC. The bond length of sp^3^ bonding on the junction line is 1.605 Å and the length of C–C bonds on the wall ranges from 1.41 to 1.52 Å, corresponding to previous report [26]. The fracture behavior occurs at tensile stain ranging from 0.075 to 0.115, corresponding to the yield stage of CHC along cell axis direction. During this period, deform mainly results from the break of C–C bonds. All the 862 C–C bonds on the junction of CHC break and then the junction is composed of an array of 8-ring cell units rather than both 5-rings and 8-rings. It is the fracture of C–C bonds on the junction that leads to the yield behavior in cell axis direction. Figure 5b presents the deformation and evolution of shear stain of local CHC structure with defect 3. Similarly, C–C bonds on the junction break at the yield stage. The C–C bond near the vacancy breaks first when CHC reaches the maximum tensile strength. Then the crash propagates rapidly leading to the failure of CHC.

### 3.3. Temperature Sensitivity of Strength of CHC with Vacancy Defects

The mechanical strength of one material is always related to temperature. To investigate the effect of temperature on mechanical properties of CHC with vacancy defect, tensile testes at the temperature of 100 K, 300 K and 500 K have been considered.

**Figure 6:**
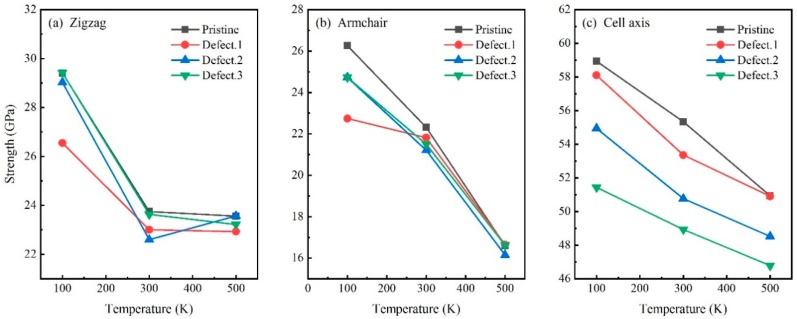
Temperature dependence. Engineering stress-strain curves of carbon honeycomb with different point defects at the temperature of 100 K, 300 K and 500 K, respectively. (**a**) zigzag, (**b**) armchair and (**c**) cell axis directions.

Figure 6 shows the temperature dependence on tensile strength of CHC with different vacancy defects. Results show that the strength of CHC decreases monotonously with the increasing temperature. The effect of vacancy defect on strength of CHC becomes more obvious when the temperature reduces. The maximum reductions of strength are 12.7% at 100 K, 4.8% at 300 K and only 2.7% at 500 K when stretching in the zigzag direction. Single point vacancy has little influence on zigzag direction at 500 K but has significant influence at 100 K. Similarly, the strength of CHC with vacancy defects in armchair direction has reduced by 9.7% at 100 K, 5.0% at 300 K and 2.7% at 500 K. Important influences of vacancy defect 1 on strength in both zigzag and armchair directions have been detected at 100 K rather than at 500 K. Similarly, the strength in cell axis direction has decreased with the increasing temperature. However, Figure 6c shows that it is vacancy defect 3 that reduce the strength of CHC in cell axis direction the most. That makes the strength in cell axis direction reduced by 12.8% at 100 K, 11.5% at 300 K and 8.1% at 500 K, respectively.

### 3.4. Effect of Vacancy Concentration on Mechanical Properties

Single atom vacancy defect will reduce the strength of CHC. Defect is evitable but the vacancy concentration may be greatly reduced by improving the synthesis conditions. Here, the effect of different number of single atom vacancy on tensile strength of CHC has been investigated. All defects are generated by removing atoms in CHC randomly. The maximum number of vacancies is 100, corresponding to 0.77% vacancy concentration. 

**Figure 7:**
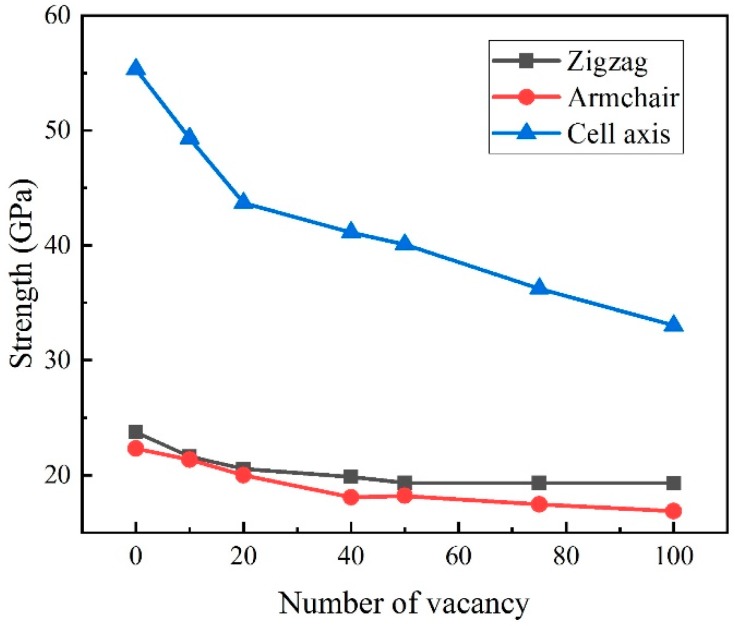
The effect on vacancy concentration on the mechanical strength of CHC for tensile loading along zigzag, armchair and cell axis direction, respectively.

Figure 7 shows the mechanical strength of CHC in different directions with different vacancy concentrations at the temperature of 300 K. The mechanical strength reduces with the increasing defect concentration. With 100 single atom vacancies, mechanical strength of CHC decreases by 18.7% in zigzag direction, 24.4% in armchair direction and 40.3% in cell axis direction, respectively. With different vacancy concentrations, the failure strain in zigzag direction ranges from 0.181 to 0.193, which is less reduction than pristine one. Similarly, failure strain in armchair direction ranges from 0.287 to 0.310. While, failure strain in cell axis direction ranges from 0.110 to 0.182. With 0.77% vacancy concentration it is only 0.110, indicating a reduction of 51% than pristine one. The failure occurs when C–C bonds on the junction is not break completely and the strength is 33 GPa which is slightly small than the yield strength of pristine one. Vacancy concentration has influence on the mechanical properties of CHC in all directions, especially for the cell axis direction. In cell axis direction, the effect of 0.77% vacancy on both failure strength and strain is critical. Previous report experimentally shows a significant drop in the mechanical properties of graphene in the vacancy-defect regime. Here, the reduction of 51% in cell axis direction shows that vacancy defect can also weaken CHC greatly [18].

## 4. Conclusions

This work investigates the mechanical properties and the effect of vacancy defect, vacancy concentration and temperature on mechanical properties of CHC. CHC yield in cell axis direction because the C–C bonds on the junction break, which changes the atomic structure of CHC. With single atom vacancy, tensile strength decreases 11.5% in cell axis direction but only around 5% in the other two directions. The position of vacancy also affects the mechanical properties in cell axis direction. Meanwhile, with defect 3 the strength of CHC reduces the most, since atoms on the wall subject to larger shear strain. Reducing temperature will increase the strength of both pristine and defective CHCs. Vacancy concentration also has influence on the mechanical properties of CHC. With 0.77% vacancy defects, the strength and failure strain in cell axis direction reduce by 40.3% and 51%, respectively. It is important for CHC that reducing the vacancy concentration by improving synthesis technology to sustain the mechanical properties.

## Figures and Tables

**Table 1:** Mechanical properties of carbon honeycomb for tensile loading along different direction.

Direction	Young’s Moduli (GPa)	Failure Strain	Strength (GPa)
Zigzag	46	0.204	23.7
Armchair	43	0.320	22.4
Cell axis	550	0.225	55.3

## Supplementary Materials

The following are available online at http://www.mdpi.com/2079-4991/9/2/156/s1, the calculation of formation energy of vacancy, Video S1: the tensile process and evolution of shear strains of CHC in zigzag direction, Video S2: the tensile processes and evolution of shear strains of CHC in armchair direction, Video S3: the tensile processes and evolution of shear strains of CHC in cell axis direction.
