# Supplementary material for: Mechanical Properties of Vacancy Tuned Carbon Honeycomb
Source: Nanomaterials (Basel). 2019 Jan 27;9(2):156. doi: 10.3390/nano9020156 (PMC6410302; doi:10.3390/nano9020156)
Supplement: Supplementary file 1 [file nanomaterials-09-00156-s001.zip › Supplementary Files.pdf]

Supplementary Information

Mechanical Properties of Vacancy Tuned Carbon Honeycomb

Lu Xie [1], Haojie An [1], Chenwei He [2], Qin Qin [1,*] and Qing Peng [3,*]

[1] University of Science and Technology Beijing, School of Mechanical Engineering, Beijing 100083, China; xielu@ustb.edu.cn (L.X.), anhaojie@xs.ustb.edu.cn (H.A.)

[2] China Nuclear Power Technology Research Institute Co., Ltd., Reactor Engineering and Safety Research Center, Shenzhen 518031, China; hechenwei@cgnpc.com.cn

[3] University of Michigan, Nuclear Engineering and Radiological Sciences, Ann Arbor 48108, MI, USA

* Correspondence: qinqin@me.ustb.edu.cn (Q.Q.), qpeng@umich.edu (Q.P.)

The Calculation of Formation Energy of Vacancy

Molecular dynamics simulations are used to calculate the formation energy of vacancy defected carbon honeycomb (CHC) using LAMMPS (64-bit 8 Mar 2018-MPI) [1]. Pristine CHC and three kind of single-atom vacancy defected CHC are used. Meanwhile, the missing atom of defect 1 is sp^3 hybridization. Defect 2 and defect 3 are on the wall of CHC with sp^2 hybridization.

The atomic interactions for C–C are described by the adaptive intermolecular reactive empirical bond order (AIREBO) potential [2]. Time step is 0.5 fs. Periodic boundary conditions are performed in all the three directions. The sizes of simulation box are 2.0 × 1.7 × 1.0 nm containing 224 atoms. The energy minimum is performed to optimize CHC structure using conjugate gradient algorithm. The isothermal-isobaric (NPT) ensemble is used to control pressure (zero) and temperature (300 K) using Nose-Hoover thermostat and barostat, respectively. The relax time is 35 ps.

The calculated total energy of pristine CHC is −1622 eV, corresponding to −7.24 eV/atom which is consistent with the result of the first-principles density functional theory calculations [3]. The total energy of three kinds of defected CHC is −1601, −1608 and −1604 eV, respectively. So, the formation energy of defect 1 is 12 eV. It is 14 eV for defect 2 and 18 eV for defect 3.

Supplementary Videos

Supplementary Video S1 shows the tensile process of CHC in zigzag direction and the evolution of shear stains. The tensile strain ranges from 0 to 0.250, and CHC fractures at 0.204 strain. The shape of the CHC hole changes from regular hexagonal edge to rectangle, and stress is majorly evenly distributed over the wall paralleled to tensile direction before failure. The tensile processes and evolution of shear strains of CHC in armchair are presented in Supplementary Video S2. The tensile strain ranges from 0 to 0.375, and structure failures at 0.320 strain. Supplementary Video S3 exhibits the tensile process and evolution of shear stains of CHC in cell axis direction. Then tensile strain ranges from 0 to 0.250, and CHC fractures at 0.225 strain. The crack triggered from a certain position propagates in multiple branches.

Reference

1. Plimpton S. Fast Parallel Algorithms for Short-Range Molecular Dynamics. Journal of Computational Physics. 1995;117(1):1-19.
2. Donald WB, Olga AS, Judith AH, et al. A second-generation reactive empirical bond order (REBO) potential energy expression for hydrocarbons. Journal of Physics: Condensed Matter. 2002;14(4):783.

3. Hu J, Wu W, Zhong C, et al. Three-dimensional honeycomb carbon: Junction line distortion and novel emergent fermions. Carbon. 2019;141:417-426. doi: 10.1016/j.carbon.2018.09.027.
